# Unlocking antitumor immunity with adenosine receptor blockers

**DOI:** 10.20517/cdr.2023.63

**Published:** 2023-10-25

**Authors:** Victoria A. Remley, Joel Linden, Todd W. Bauer, Julien Dimastromatteo

**Affiliations:** ^1^Department of Surgery, University of Virginia, Charlottesville, VA 22903, USA.; ^2^Adovate, Charlottesville, VA 22901, USA.; ^3^University of Virginia Comprehensive Cancer Center, Charlottesville, VA 22903, USA.

**Keywords:** Immunotherapy, adenosine, adenosine receptors, adenosine A2A receptors (A2AR), adenosine A2B receptors (A2BR), tumor cells, immune cells, tumor microenvironment

## Abstract

Tumors survive by creating a tumor microenvironment (TME) that suppresses antitumor immunity. The TME suppresses the immune system by limiting antigen presentation, inhibiting lymphocyte and natural killer (NK) cell activation, and facilitating T cell exhaustion. Checkpoint inhibitors like anti-PD-1 and anti-CTLA4 are immunostimulatory antibodies, and their blockade extends the survival of some but not all cancer patients. Extracellular adenosine triphosphate (ATP) is abundant in inflamed tumors, and its metabolite, adenosine (ADO), is a driver of immunosuppression mediated by adenosine A2A receptors (A2AR) and adenosine A2B receptors (A2BR) found on tumor-associated lymphoid and myeloid cells. This review will focus on adenosine as a key checkpoint inhibitor-like immunosuppressive player in the TME and how reducing adenosine production or blocking A2AR and A2BR enhances antitumor immunity.

## INTRODUCTION

Deadly tumors have the ability to resist the body’s formidable immune defenses. They create protective micro-environments that limit antigen presentation, inhibit T and natural killer (NK) cell responses, and induce T cell exhaustion, effectively escaping immune surveillance. These mechanisms allow some tumors to grow unchecked and resist conventional cancer therapies.

Checkpoint inhibitors that block immunosuppressive signaling molecules such as PD-1, T lymphocyte antigen 4 (CTLA4), and lymphocytic activation gene 3 protein (LAG-3) have ushered in a new era of cancer immunotherapy, offering hope for prolonged survival and enhanced quality of life for many patients. However, the beneficial effects of these therapies are not universal due to the ability of some tumors to maintain an immunosuppressive environment. The interplay between cancer cells and immune cells within the tumor microenvironment (TME) is a critical determinant of the therapeutic response.

A key driver of immunosuppression within the TME is extracellular adenosine (ADO), an adenosine triphosphate (ATP) metabolite. ADO formation and its immunosuppressive signaling play a pivotal role in maintaining the immunosuppressive state of the TME, promoting tumor growth, and facilitating resistance to other therapies. This review explores the role of ADO signaling in the TME. Inhibiting ADO receptors on immune cells reduces immunosuppression and, in some cases, has an additive antitumor effect when combined with other cancer treatments.

## UNDERSTANDING NON-RESPONDERS TO IMMUNOTHERAPY IN SOLID TUMORS

Despite having antitumor effects, cancer immunotherapy often fails. One prominent reason is that most tumor proteins are recognized as self-proteins and fail to activate T cells, which serve as the frontline warriors of the adaptive immune response^[1]^. To the extent that tumors are recognized by the immune system, their activation is muted by immunosuppressive signals like adenosine.

Antigen-presenting cells (APCs) such as macrophages and dendritic cells (DCs) also regulate the immune response to tumors^[2]^. The function of APC is to recognize, engulf, and present tumor antigens on their surface. Their failure to optimally phagocytose and present antigens can undermine the initiation of adaptive immunity. The host’s baseline immune response also significantly influences the success or failure of cancer immunotherapy. The baseline immune response is crucial in determining treatment efficacy, represented by the association between increased T cell infiltration into tumors and improved patient survival and immunotherapy response rates^[3]^. However, the factors that dictate the extent of T cell infiltration into tumors, whereby an extensively infiltrated tumor is considered “hot”, and a sparsely infiltrated tumor is considered “cold”, are just beginning to be elucidated. The factors that influence infiltration vary across tumor types and subtypes due to immune cell heterogeneity^[4]^. The complexity and dynamics of the immune system, in conjunction with the adaptability of tumor cells, create a challenging landscape for the successful deployment of cancer immunotherapy.

### The complexity of the TME in solid tumors

The TME in solid tumors is complex, consisting of various immune cells, cytokines, chemokines, and metabolites. Specific features of the TME depend on the tumor type and the location within the patient. Some tumors develop an extracellular matrix (ECM) of fibrous proteins and stromal cells that define and isolate the TME from the surrounding tissue^[5,6]^. Within “cold” solid tumors, very few antitumor CD8+ T cells, NK cells, and DCs are present, due to failure by immune cells to enter through the ECM^[7]^. Immune cells that contribute to the immunosuppressive state are tumor-associated macrophages (TAMs) and myeloid-derived suppressor cells (MDSCs). Monocytes that enter tumors can polarize into M1 (proinflammatory) or M2 (immuno-suppressive) cells. In cancer, most become M2 and MDSC^[8]^ and function to secrete vascular endothelial growth factor (VEGF) and transforming growth factor β (TGF-β), which stimulates angiogenesis within the tumor^[9]^. M2-TAMs and MDSCs also suppress CD8+ T cell infiltration and increase regulatory T cell (Treg) differentiation from precursor CD4+ T cells^[10]^. Tregs release suppressive cytokines such as interleukin-10 (IL-10) and TGF-β that inhibit CD8+ T cell function and enhance cancer cell escape from the immune attack^[11]^. In some solid tumors, such as pancreatic ductal adenocarcinoma (PDAC), increased IL-10 is correlated with reduced survival^[12-14]^.

MDSCs are considered immature myeloid cells and secrete reactive oxygen species (ROS), IL-10, IL-13, TGF-β, arginase-I, inducible nitric oxide synthase (iNOS), and other immunosuppressive factors^[15-17]^.

The TME is usually hypoxic due to a poorly developed tumor vasculature. Most cytotoxic immune cells (CD8+ T cells and NK cells) function poorly in hypoxic states, while suppressive cells (M2 TAMs and Tregs) thrive^[18]^. The hypoxic TME favors the production of ADO and the induction of immunosuppressive A2A receptors (A2AR) and A2B receptors (A2BR) in immune cells. A shift in tumor metabolism from oxidative phosphorylation to primarily glycolysis also suppresses immune cell infiltration due to increased lactate in the TME. The increase in lactate lowers tumor pH^[19]^. This decrease in pH drives M2 polarization while inhibiting the nuclear factor of activated T cells (NFAT) within T cells^[20,21]^. This suppression of NFAT inhibits chemotaxis into tumors and reduces T cell activation.

### The role of immune checkpoint inhibitors (ICIs) in cancer immunotherapy

Immune checkpoint inhibitors (ICIs) have greatly advanced immunotherapy, especially in some hard-to-treat tumors. The two most studied checkpoints are CTLA4 and PD-1^[1,22,23]^. Although antibodies that block these inhibitory signals have improved survival, most patients with solid tumors eventually develop either primary or secondary resistance to ICI. In primary resistance, tumors display early resistance to ICI and progress soon (within six months) after ICI treatment. In secondary resistance, patients respond to treatment initially but develop resistance later^[1]^. Studies have demonstrated that the tumor mutational burden (TMB) influences the response to ICI^[24-26]^. Reduced TMB within tumors treated with ICI can result in acquired resistance to ICI immunotherapy.

### Contributions to therapy resistance by suppressive state and hypoxia

Suppressive immune cells within the TME contribute to checkpoint inhibitor resistance. Tregs, MDSCs, and M2-TAMs secrete immunosuppressive cytokines (TGF-β, CXCL8, CCL5, and IL-10) that prevent cytotoxic infiltrating immune cells from entering the tumor^[27-31]^. An increase in VEGF due to the activation of the mitogen-activated protein kinase (MAPK) pathway can also stimulate tumor angiogenesis^[27-31]^ and inhibit immune cell infiltration^[32]^.

Hypoxia results from various physiological and pathological conditions, including solid tumors, ischemia-reperfusion injury, stroke, and chronic obstructive pulmonary disease (COPD)^[33]^. A hypoxic TME contributes to an increase in extracellular ADO. The molecular mechanisms underlying hypoxia-driven responses include *Adora2a* and *Adora2b* via HIF-1α and HIF-2α^[34,35]^. HIFs also stimulate angiogenesis, vasodilation, and attenuation of inflammation^[34,36]^. HIF-1α induces CD73 and CD39 and increases the conversion of ATP into ADO, leading to T cell inhibition, metastasis, and increased angiogenesis^[37-40]^. The accumulation of ADO within tumor suppresses cytotoxic immune cells and APCs, such as CD8+ T cells and DCs, while enhancing the accumulation of immunosuppressive cells^[41,42]^. When ADO encounters its receptors, it can affect the activity of neutrophils and macrophages, reducing the release of IL-12, tumor necrosis factor-alpha (TNFα), and ROS^[43-45]^.

### The role of cancer-associated fibroblasts (CAFs) in tumor development

Cancer-associated fibroblasts (CAFs) have a different morphology than other cells within the tumor. They lack epithelial, endothelial, and leukocyte markers, and do not have the same mutations as tumor cells^[46]^. CAF development within tumors occurs when there is an increase in inflammatory markers such as IL-6 and TGF-β due to cancer cell DNA damage^[47,48]^. When IL-6 and TGF-β are increased in the TME, they tend to reduce the number of T cells, limiting the extent of the antitumor response^[49]^. These cytokines increase JAK-STAT signaling and ECM transition, promoting CAF formation^[50-52]^. Breast cancers increase Notch signaling within the TME to increase CAFs^[53]^. Patients who receive chemotherapy and radiation to treat solid tumors experience DNA breaks, and this stress can promote fibrosis or CAF accumulation and function. This change in CAF function causes resistance to therapy in various solid tumors^[54-56]^.

Solid tumor CAFs may exhibit different phenotypes depending on the TME. The different phenotypes exhibit different cell surface markers, but identifying these can be challenging^[46]^. Breast cancers increase expression of fibroblast activation protein (FAP) to cause high immunosuppression through Treg activation^[57]^. Pancreatic cancers express both myofibroblastic CAFs (myCAFs) and inflammatory CAFs (iCAFs) at different locations in the tumor. MyCAFs have high expression of TGF-β and αSMA and are located close to tumor cells, while iCAFs have high IL-6 secretion and are more distal in the TME^[58,59]^. iCAFs can recruit TAMs and MDSCs to the TME to increase the immunosuppressive state^[13,60]^. Targeting the MAPK/STAT pathways in iCAFs through inhibitors in combination with checkpoint inhibitor therapy (e.g., anti-PD-1) can lead to increased survival of patients with solid tumors like PDAC^[60]^. McAndrews *et al.* discovered that in early-stage PDAC, iCAFs tend to be more abundant, while myCAFs have a higher abundance in late-stage cancer. When FAP+ CAFs were depleted, there was an increase in mouse survival. Conversely, when αSMA+ CAFs were depleted, there was a decrease in survival. In the TME, when FAP+ CAFs were depleted, there was a decrease in macrophages and B cells. However, αSMA+ CAF loss showed a decrease in effector T cells (Teff) and increased Tregs. A loss in IL-6 production in FAP+ CAFs increased responses in mice to gemcitabine therapy and combination therapy of gemcitabine + checkpoint inhibitors^[57]^.

## THE ADENOSINE PATHWAY: A NEW APPROACH TO OVERCOMING THERAPEUTIC RESISTANCE TO CHECKPOINT INHIBITORS

Understanding the interaction between cells in the TME is crucial to overcoming therapeutic resistance. The concept of targeting ADO biosynthesis or inhibiting its receptors has garnered increased interest from the scientific community^[58]^. Targeting extracellular ADO and its receptors opens opportunities for increasing the antitumor immune response in innate and adaptive immune cell populations normally suppressed within the TME^[59]^. Since high ADO levels in the TME are predictive of immunosuppressive responses^[60]^, combining current immunotherapies with ADO blockade may help to overcome ICI resistance in solid tumors.

Chemotherapies and various cancer treatments result in elevated cell death and heightened ATP release^[61]^. ATP is rapidly converted to ADO within solid tumors. This process is mediated by ectonucleotidases CD39 and CD73 and contributes to the formation of an immunosuppressive TME^[62]^. CD39 acts on ATP to produce adenosine monophosphate (AMP), which is subsequently converted into ADO by CD73. The resulting extracellular ADO interacts with one of four G-protein-coupled receptors (A1R, A2AR, A2BR, and A3R) found in tumor cells, immune cells, and endothelial cells. This increase in ADO levels within the TME hinders the activity of effector immune cells and promotes the expansion of immunosuppressive regulatory T cells^[63]^. Exosomes released into the TME during cell death also express CD73 and CD39^[62]^.

The A2AR is associated with elevated levels of checkpoint molecules like PD-1, CTLA4, and LAG-3 on T cells^[64]^. Activation of this receptor tends to inhibit the antitumor functions of macrophages and the proliferation and cytokine production of cytotoxic T cells^[65]^. However, increased expression of CD39 and CD73 within the TME leads to an upsurge in MDSCs and Tregs^[59]^.

### The influence of adenosine receptor expression in key immune cells

#### Innate immunity: the role of adenosine receptors in macrophages, dendritic cells, and natural killer cells

**Figure 1:**
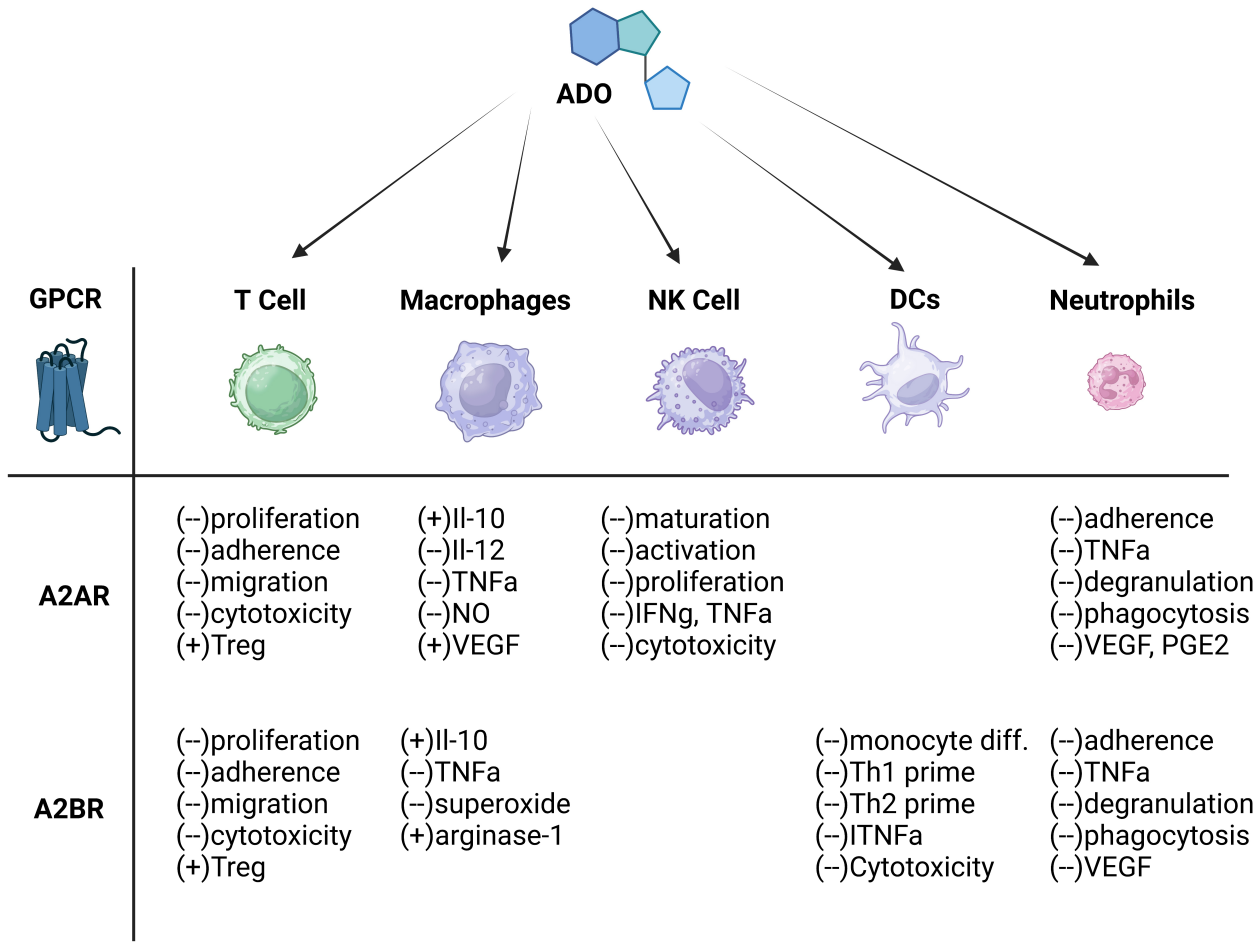
Adenosine’s pleiotropic effects on immune cells. ADO facilitates the evasion of tumor cells from immune detection by restricting the activity of T cells, DCs, NK cells, macrophages, and neutrophils. Concurrently, adenosine amplifies the functionality of immunosuppressive cell types like MDSCs and Tregs. ADO: Adenosine; A2AR: A2A receptors; A2BR: A2B receptors; DCs: dendritic cells; MDSCs: myeloid-derived suppressor cells; NK: natural killer; TNFα: tumor necrosis factor-alpha; Tregs: regulatory T cells; VEGF: vascular endothelial growth factor.

Innate immunity plays a critical role in the body’s defense against cancer by providing the first line of protection against malignant cells [Figure 1]. This system, comprising various immune cells such as NKs, TAMs, DCs, and soluble factors like cytokines and chemokines, acts to identify and eliminate transformed cells. Recent studies have highlighted the dynamic interplay between innate immunity and cancer progression, shedding light on the delicate balance between tumor-promoting and tumor-suppressing functions of the innate immune system. An essential aspect of the innate immune system’s interaction with cancer cells involves the A2BR, which modulates immune cell functions and significantly impacts the balance between tumor-promoting and tumor-suppressing activities.

#### Tumor-associated macrophages

TAMs exhibit substantial plasticity that can play a part in tumor progression and drug resistance^[66]^. The two main classes of TAMs within the TME are activated M1 and alternatively activated M2^[67]^. M1 is known to be the proinflammatory subset within the tumor, while M2 is considered to be suppressive. However, the classes are not static; the cells can change their state based on the cytokines present. There are also subsets of M2 within the TME, and each class plays a role in tumor formation and progression^[67,68]^.

The TME regulates M1 and M2 macrophages to regulate the immune response to tumors. TAM precursors are derived from embryogenic or bone marrow-derived monocytes^[66]^. TAMs tend to differentiate primarily into M2-like phenotypes. These cells express high levels of VEGF (pro-angiogenic), mannose receptor (CD206), and scavenger receptor (CD163). They release suppressive cytokines such as IL-10 and promote immunosuppression within the tumor. M1 plays a key role in vaso-proliferation through the secretion of inflammatory cytokines such as IL-6, IL-8, TNF-α, and IL-1β^[67,69,70]^. The presence of TAMs with high levels of IL-1β within the TME contributes to neovascularization and is a predictor^[69]^.

Apoptotic cells phagocytosed by TAMs (aka efferocytosis) release ATPs into the extracellular space in tissues^[71]^. ATP derivatives, specifically ADO, affect the immune activation of TAM through ADO receptors^[71-73]^. The A2BR is upregulated on TAMs in response to interferon-gamma (IFN-γ). When activated, A2BR suppresses the production of TNFα in infiltrating TAMs, inhibiting their capacity to secrete cytokines that are crucial for antitumor immunity. This process ultimately promotes tumor growth^[71]^.

#### Dendritic cells

DCs are important APCs that present antigens to T cells on MHC proteins. T cells that recognize self-proteins abundant in tumors do not survive selection in the thymus. Intra-tumoral injections of DCs that initiate CD8+ T cell activation have been used to increase responses to immune checkpoint blockade immunotherapies^[74-76]^. Damage-associated molecular patterns (DAMPs) are used to determine if an immune response needs to be stimulated or if there is immune tolerance to that antigen^[74,77]^. DAMPs initiate a CD8+ T cell response within tumors but can also help re-prime effector CD8+ T cells to continue the adaptive immune response. However, when tumor cells overcome immune surveillance, DCs may have altered antigen processing and defective T cell activation^[78]^.

Within the TME, there are numerous subsets of DCs along with migratory/tissue-resident DCs^[79]^. Classical DCs are derived from common myeloid progenitors that differentiate into common DC progenitors. Plasmacytoid DCs (pDC) are believed to be derived from lymphoid cells but can also be derived from myeloid precursors. Common monocyte precursors differentiate into a third major subtype of DCs^[80]^. Classical DCs have two major states: type 1 and type 2 (cDC1 and cDC2). cDC1 acts to recognize apoptotic and necrotic cell debris presented on its MHC-I receptors to activate CD8+ effector T cells. Their function helps to drive an antitumor response within the TME^[75]^. cDC2 are more heterogenous than cDC1 cells in tumors but are believed to play a role in recognizing exogenous tumor antigens and presenting them to CD4+ T cells on MHC-II^[81]^. Within cDC2 populations, there are two further subtypes: anti-inflammatory (cDC2A) and proinflammatory (cDC2B). Classical DCs are now emerging as a potential target for PD-1/PD-L1 immune checkpoint blockade. It has been demonstrated that the proper functioning of checkpoint blockade requires cis interactions with CD80 and PD-L1, as well as PD-1 and PD-L1, between T cells and the DCs^[82,83]^.

DCs that have differentiated during exposure to ADO display diminished activity. Moreover, these DCs express high levels of angiogenic, immunosuppressive, proinflammatory, and tolerogenic factors, such as cyclooxygenase-2 (COX-2), indoleamine 2,3-dioxygenase (IDO), interleukin-6 (IL-6), interleukin-8 (IL-8), IL-10, TGF-β, and VEGF^[84,85]^. These DCs depend on the upregulation of A2BR when producing these factors to promote ongoing tumor growth and increased angiogenesis for metastasis^[85]^. As a result, blocking A2BR can preserve the activity of DCs to present neoantigens to T cells, thereby facilitating the process of tumor cell destruction.

#### NK cells

NKs are derived from CD34+ hematopoietic stem cell progenitors in bone marrow. These cells kill targets that express either no or extremely low MHC-I on their surface^[86,87]^. NK cells have a specific killer immunoglobulin-like receptor (KIR) on their surface that recognizes MHC-I molecules. When the KIRs recognize MHC-I in self-cells, NK cells are downregulated to prevent an immune response^[86,88,89]^. Tumor cells tend to have low MHC-I, which can stimulate suppressed NK cells to become activated^[90]^. NK cells often recognize specific cancer ligands upregulated within tumors, such as MHC-I polypeptide-related sequence A (MICA), MICB, UL-16 binding proteins, complement factor P, and platelet-derived growth factor DD^[91-93]^.

NK cells may have an important role in tumor immunosurveillance. In some cancers, such as colon cancer and gastrointestinal stromal tumors, low NK cell expression is associated with poor outcomes^[94,95]^. NK cells can also kill circulating tumor cells that are implicated in metastasis^[96]^. Tumors may become resistant to NK cell effects by suppressing immune cell activation. Like other immune cells, the hypoxic and low-nutrient environment in tumors can decrease NK cell activation^[97]^. Tumors increase the interactions of activating ligands with their receptors on NK cells to stimulate later resistance to the cells. This increased stimulation can suppress the NK cell function^[98]^.

When NK cells are activated and encounter adenosine through the A2BR, it triggers the cyclic adenosine monophosphate (cAMP) pathway. This activation subsequently blocks cytotoxic activity and cytokine production, diminishing antitumor activity^[99,100]^. NK cells can also increase CD73 on their surface after encountering mesenchymal stromal cells, thereby contributing to an increase in ADO and tumor growth^[101]^. This increase in ADO associated with high levels of A2AR contributes to suppressing NK antitumor function^[102,103]^.

#### Adaptative immunity - the influence of adenosine receptors on t cell function

Adaptive immunity is the second line of defense in the immune system during infection or cancer. These immune responses are cytotoxic to tumor cells. The main two tumor-infiltrating lymphocyte (TIL) cell populations that mediate adaptive immunity are T cells (CD4+ and CD8+) and B cells. T cell infiltration within tumors depends on the tumor chemokine profile and how easily immune cells can enter through the tumor extracellular matrix. Over the last decade, improved methods have been developed to engineer T cells to be better at avoiding cancer immunosuppression. These techniques have resulted in clinical trials of blood-derived tumors and some sarcomas. Very little progress has been made for solid tumors. However, targeting the A2AR on T cells may help overcome the difficulties of T cell immunotherapies in solid tumors. Since ADO causes an increase in Tregs among infiltrating T cells, blocking A2AR can help maintain a high amount of CD4+ T cells in solid tumors. Relative to T cells, B cells have very low levels of A2BR, and limited studies have investigated its role in A2BR activation from ADO^[104]^. A2AR are more abundant in human than mouse B cells, but their role in immunotherapy is unknown.

#### T cells

T cells have been the main target of immunotherapy. CAR T cells and immune checkpoint inhibitors have been used to enhance T cell-mediated tumor killing. Dangaj *et al.* demonstrated that CCL5 must be present within the TME for TILs to enter solid tumors. The macrophages and DCs within the tumor also need to produce CXCL9 to aid in T cell infiltration^[105]^. Anti-PD-1 therapies have only shown limited success in solid tumors. In metastatic head and neck squamous cell carcinoma, only 15% of patients responded to anti-PD-1 treatment, and very few responses have been seen in microsatellite-stable colorectal cancer^[106,107]^. Duhen *et al.* discovered a subset of CD4+ T cells in tumors that are double positive for PD-1 and inducible costimulator (ICOS)^[106]^. These cells can have a tumor tissue-resident phenotype that allows them to recognize both tumor antigens and neoantigens on MHC-II. CD8+ TILs, on the other hand, are more heterogeneous in their response to tumor antigens^[108]^. The presence of PD-1 and ICOS on CD4+ T cells may work in conjunction with CD8+ T cells to stimulate a robust antitumor response.

Co-expression of CD39 and CD103 on CD8+ TIL within solid tumors has shown promise in targeting tumor cells. CD8+ T cells that have high expression of CD39 and CD103 can be identified in both primary tumors and metastatic sites but not in the periphery^[109]^. The level of CD39 and CD103 double positive (DP) cells determines how well patients will respond to immunotherapy^[110,111]^. However, tumors can still escape these DP TILs through exhaustion mechanisms. All DP TILs express high levels of PD-1 and other exhaustion markers^[112]^. Checkpoint inhibitors may be useful, but there are also additional ADO pathways linked to CD39+ cells. Blocking the ADO pathway and using immune checkpoint inhibitors may help keep the DP CD8+ T cells active and prevent tumor growth.

Tumor cells increase their expression of CD39 to suppress both CD4+ and CD8+ T cell proliferation and cytotoxicity in the TME^[100]^. Activation of A2AR in T cells causes increased CD4+ differentiation into Treg cells. There is also an increase in additional suppressive receptors such as PD-1, LAG-3, CTLA4, and T cell immunoglobulin and mucin domain-containing protein 3 (TIM3) on the T cells. Increases in ADO may have a negative effect on immunotherapies when checkpoint inhibitors are given to patients alone^[113]^.

### The importance of ADO biosynthesis in the TME

**Figure 2:**
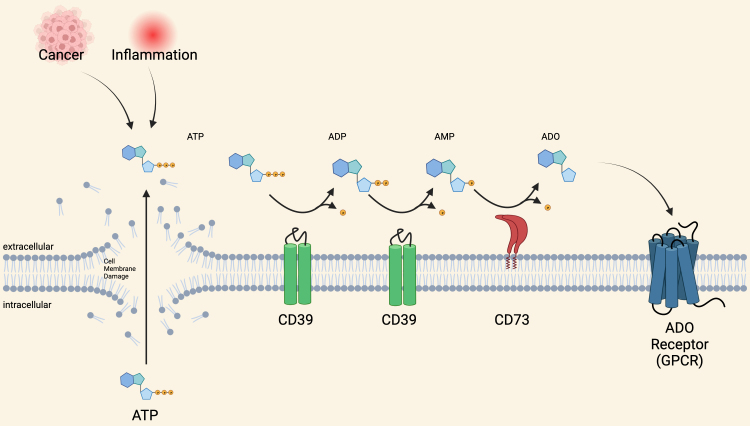
Adenosine Biosynthesis in Inflamed Tissues. The accumulation of extracellular ATP, driven by stress-induced conditions, stimulates extracellular ADO production by the enzymes CD39 and CD73. ADO binds four G protein-coupled receptors, A1, A2A, A2B, and A3. ADO: Adenosine; ADP: adenosine diphosphate; AMP: adenosine monophosphate; ATP: adenosine triphosphate.

Extracellular ADO is found at low levels in unstressed tissues^[114]^. It is produced in response to the breakdown of adenine nucleotides and AMP outside injured cells [Figure 2]^[115]^. In response to cancer initiation, ADO levels rapidly increase within tissues due to hypoxic, inflammatory, and/or ischemic conditions. Stressed cells release ATP into the extracellular space as a distress signal that transiently signals via P2 purinergic receptors. Ectoenzymes CD39 and CD73 can rapidly break down extracellular ATP on cell surfaces to produce extracellular ADO^[116,117]^. Initially, CD39 converts ATP into adenosine diphosphate (ADP) and AMP, followed by CD73-mediated conversion of AMP into ADO^[118]^. The accumulation of ADO in the TME helps create the suppressive niche.

#### Inhibition of critical immune mechanisms stimulates the formation of the pro-adenosine niche and fibrotic remodeling

The formation of solid tumors in tissues begins with an increase in cell death, inflammation, and hypoxia. This leads to an increase in extracellular ATP and ADO within the TME. When the proinflammatory metabolite extracellular ATP is cleaved into extracellular ADO and is recognized by the A2AR and A2BR within tumors, there is suppression of immune functions^[59]^. Endothelial cells within the forming tumor and infiltrating immune cells express CD39 and CD73 on their surface. This allows for an increase in ADO within the TME. Endothelial and immune cells also express A2BR on their surface, and when activated by ADO, the tumor can suppress immune cell infiltration. Solid tumors become hypoxic, which feeds back to increase ATP, CD73, and CD39 in the TME to further suppress the immune infiltration^[115]^. Tumor cells interact with suppressive immune cells to increase A2BR expression, leading to metastasis, proliferation, and VEGF production^[119]^.

CAF increases within solid tumors, forming a dense tumor stroma. These CAFs express high levels of CD39 and CD73 on their surface in various solid tumors such as ovarian, pancreatic, colorectal, and breast cancer, which contribute to ADO production^[120-122]^. A dense fibrotic stroma allows ADO to remain in high concentration to drive immunosuppressive signaling throughout the tumor. An increase in A2BR on CAFs increases the secretion of IL-6 into the TME, which can convert epithelial cells to a more mesenchymal phenotype^[63]^. This remodeling of the TME leads to increased metastasis and therapy resistance.

#### Increases in the ADO pathway cause resistance to immune checkpoint inhibitor therapies

Immune checkpoint inhibitors such as anti-PD-1 and anti-CTLA4 have shown great promise for improving the survival of patients with solid tumors. This form of therapy targets PD-1 and CTLA4 on CD8+ T cells. Tumor cells inhibit CD8+ T cell function by targeting these checkpoint molecules. By blocking PD-1 and CTLA4 from being recognized, the cytotoxic function of the CD8+ T cells is increased to clear the tumor cells^[113]^. However, patients with solid tumors tend to relapse and become resistant to checkpoint therapy. Maj *et al.* discovered that when checkpoint therapy is given, there is an increase in the death of cancer cells and Tregs. The sudden death of these cells releases a high amount of ATP in the TME, which is then converted to adenosine by CD39 and CD73. This increase in ADO in the TME counteracts checkpoint therapy and suppresses the antitumor immune response^[123]^.

While an increase in CD73 and CD39 is associated with poor prognosis in patients, increasing A2AR and A2BR expression also contributes to an increased risk of resistance to checkpoint therapy. It has been found that having an increase in A2AR in non-small cell lung cancer (NSCLC) or A2BR in triple-negative breast cancer (TNBC) contributes to poor survival^[119,124]^. Chalmin *et al.* showed that the adenosine pathway is involved in resistance to anti-PD-1 therapies. They demonstrated that when patients were given checkpoint therapy, there was an increase in CD73, leading to resistance^[125]^. Combination therapies targeting CD73, and checkpoint inhibitors may help overcome early resistance in solid tumors. Studies have shown that targeting CD73 and PD-1 in murine colon tumors can inhibit tumor growth^[126]^. The idea that targeting both the adenosine pathway and checkpoint inhibitors may overcome resistance in solid tumors gives promise to advancing immunotherapy.

#### Endothelial cells increase CD39 and CD73 levels during hypoxia in tumors, leading to angiogenesis

**Figure 3:**
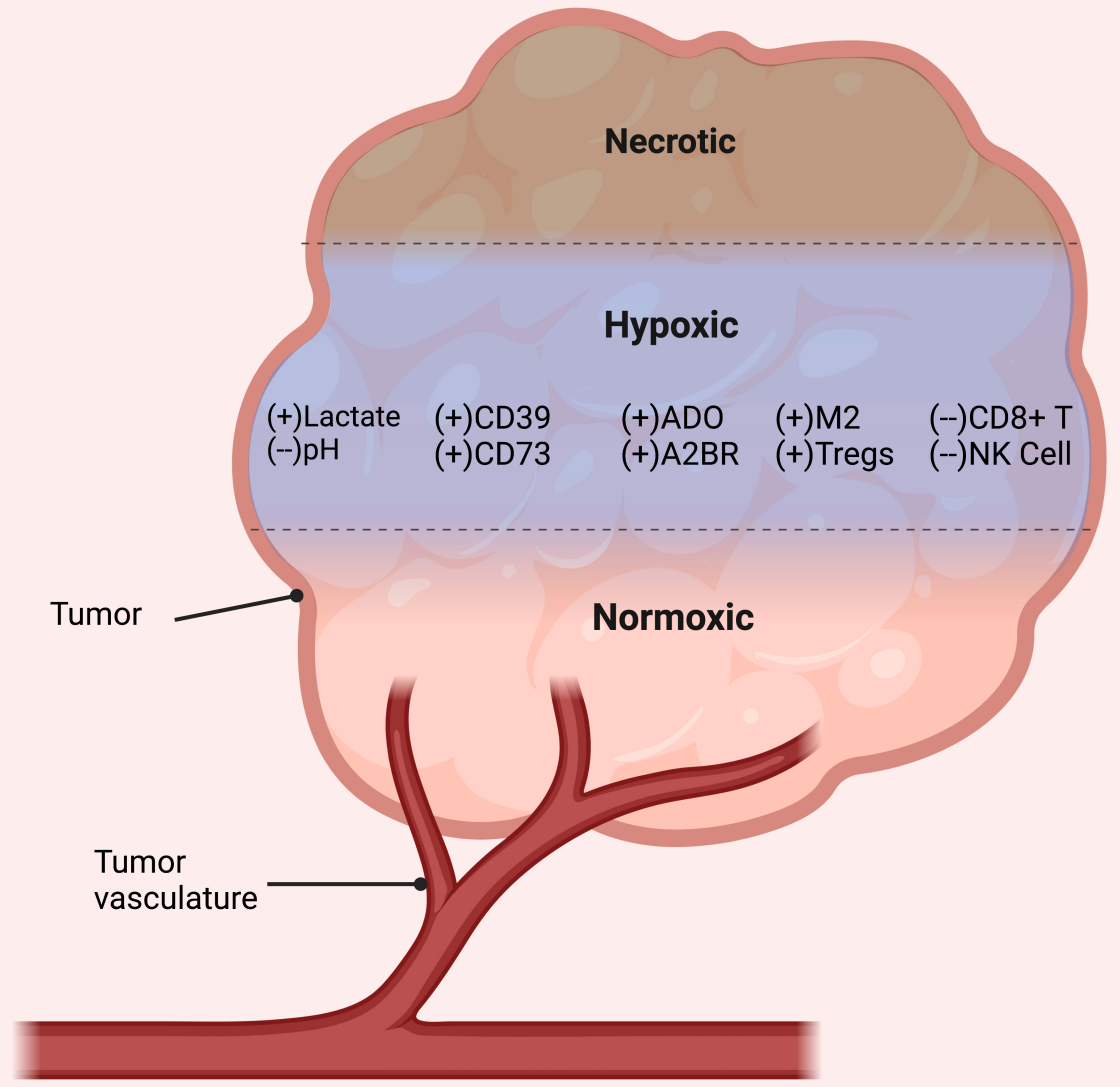
Hypoxia in the TME. This figure illustrates a region of a tumor devoid of vasculature, leading to an oxygen-starved environment. This hypoxic zone is characterized by low pH and a predominance of lactate. Within this environment, TAMs tend to adopt an M2 phenotype, and Tregs become the predominant T cell population. Hypoxic conditions also encourage the expression of the ectoenzymes CD39 and CD73, leading to a surge in extracellular ADO. This increase in adenosine, in turn, helps to sustain an immunosuppressive environment within the tumor. Note: this illustration does not depict the anatomical structure of the tumor, but rather represents the phenomena occurring at different levels of tumor oxygenation. ADO: Adenosine; A2BR: A2B receptors; NK: natural killer; TAM: tumor-associated macrophage; TME: tumor microenvironment; Tregs: regulatory T cells.

Hypoxia occurs in solid tumors and suppresses immune cell infiltration by activating hypoxia-inducible factor 1/2 (HIF1/2), IL-6, TGFβ, and TNF. Under these conditions, endothelial, tumor, and various suppressive immune cells increase CD73 and CD39 to increase ATP conversion to AMP and ADO^[39,127]^. Tumor cells that increase CD73 expression can generate ADO to interact with A2ARs on the tumor cells to stimulate an increase in VEGF secretion^[128]^. VEGF works to increase angiogenesis within the tumor and provides nutrients and oxygen for growth and metastasis [Figure 3].

Endothelial cells express CD39 within tumors to degrade ATP and promote increased neovascularization and tumor growth^[129]^. To have the ADO concentration needed to sustain the tumor-protective endothelial barrier, CD73 and A2BR are needed within the TME^[130]^. Feng *et al.* and Sun *et al.* demonstrated that inhibiting CD39 on solid tumor endothelial cells decreased angiogenesis and tumor growth^[129,131]^. CD39 on endothelial cells and the vascular is highly expressed within pancreatic and rectal carcinoma. High expression of these cells in these tumors is correlated with early TNM and better survival after tumor resection^[132,133]^. Studies have shown that having high levels of CD73 in many solid tumors is associated with worse outcomes. High CD73 levels correlate with higher adenosine concentrations in the tumor, leading to a sustained immunosuppressive TME. With a high expression of both CD39 and CD73 within solid tumors, future combination therapies targeting CD39/CD73, PD-1/CTLA4, and A2BR may allow for better survival in patients.

### ADO receptors play various roles in cancer growth

**Figure 4:**
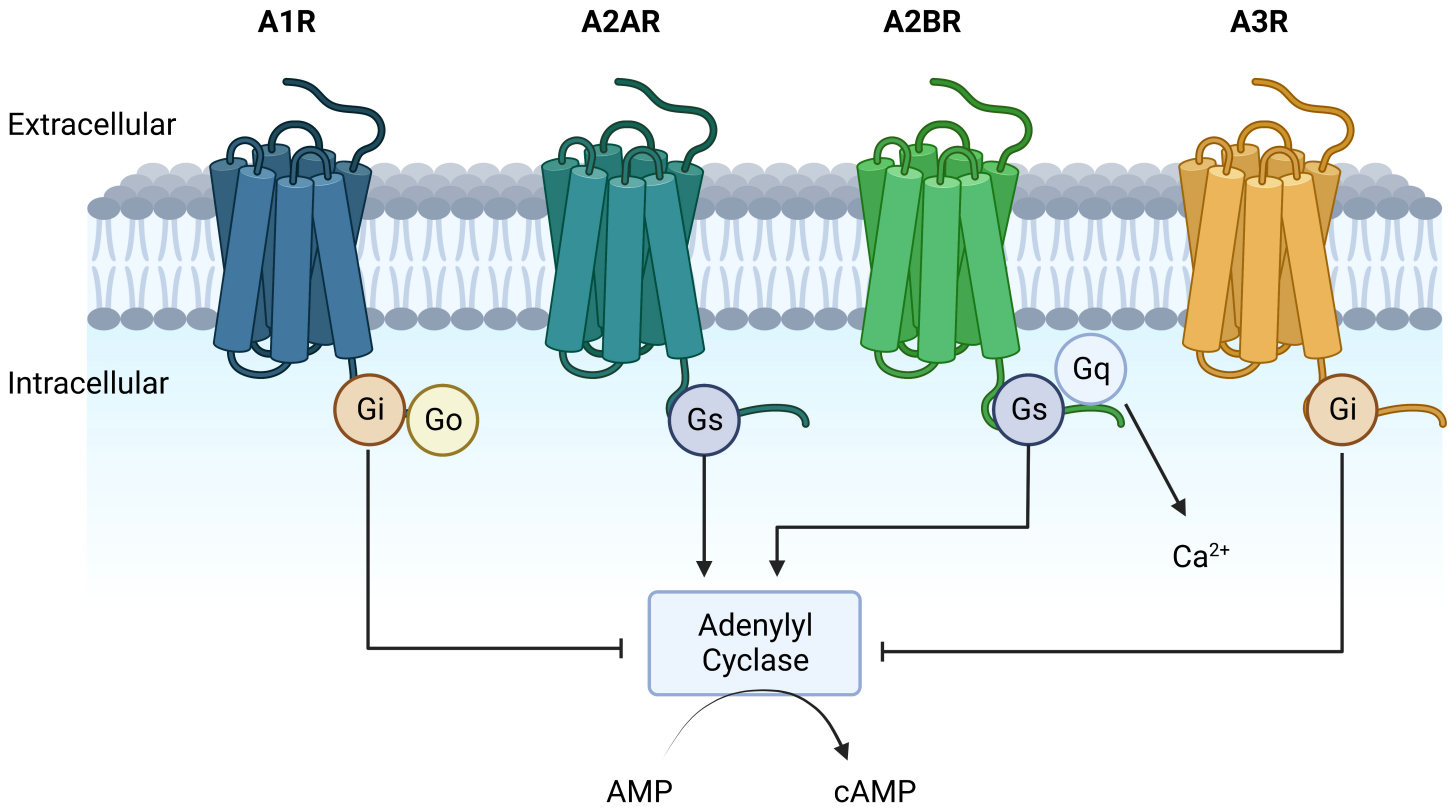
Adenosine receptors. Adenosine interacts with four distinct receptors - A1R, A2AR, A2BR, and A3R. Each of these receptors is linked to a G protein-coupled receptor. The A1R and A3R couple with the Gi protein to inhibit Adenylyl Cyclase. In contrast, the G protein coupled to A2AR and A2BR activates Adenylyl Cyclase, leading to an increased formation of intracellular cyclic AMP. A2BRs also couple to Gq which mobilizes Ca^2+^ upon activation. AMP: Adenosine monophosphate; A2AR: A2A receptors; A2BR: A2B receptors; cAMP: cyclic adenosine monophospha.

An increase in adenosine within the TME allows for immunosuppression that promotes tumor growth. Receptors for ADO on tumor cells, endothelial cells, and immune cells are drivers of tumor growth and metastasis. The ADO G-protein coupled receptors have four subtypes: A1, A2A, A2B, and A3 [Figure 4]. The A1R, A2AR, and A2BR proteins are highly conserved, while the A3R varies among species. These receptors interact with MAPK pathways to promote proliferation. A2AR and A2BR also increase activation of the mTOR and ERK pathways^[134]^. However, receptor signaling is dependent on the concentration of extracellular ADO. This level of ADO is primarily dictated by ATP and ADP metabolism by CD39 and CD73 on cells to make AMP and then ADO. In the following, we will focus only on A2AR and A2BR, which appear to play major roles in tumor immunosuppression.

#### A2AR and A2BR increase immunosuppression in solid tumors

Both A2AR and A2BR are involved in ADO’s immunosuppressive functions. A2AR and A2BR are expressed in most immune and tumor cells^[59,135]^. A2BR on myeloid cells normally has lower expression than A2AR on other immune cells. However, this receptor increases in the presence of pathological responses such as infections or cancer^[136]^. A2BR upregulation and activation signal macrophages towards the suppressive M2-like phenotype and secrete IL-10 and VEGF to promote angiogenesis and tumor growth^[43]^. A2ARs on infiltrating T cells within the TME are activated when ADO increases in the surroundings. This activation suppresses the effector CD8+ T cells in the TME while signaling CD4+ T cell differentiation into Tregs^[137-139]^. IFN-γ production decreases in NK cells when the A2AR on these cells is activated by ADO^[99]^. Decreases in antitumor cytotoxic cells and cytokines allow for increased suppressive factors that drive tumor growth and resistance to antitumor therapies.

A2BR activation due to TME hypoxia works to maintain the epithelial barrier of the tumor^[39]^. Maintaining this barrier prevents antitumor immune cells from penetrating the tumor. A2AR and A2BR-mediated immunosuppression also allow for an increase in metastasis amongst solid tumors^[58]^.

#### A2BR is key for immunosuppression in solid tumors caused by ADO

Since A2BRs are expressed at low levels under normal conditions, this receptor may be key for triggering immunosuppression within solid tumors. The A2BR has the lowest potency for ADO under normal physiological conditions. However, during inflammation and sudden increases in apoptosis, the A2BR is activated to create an immunosuppressive niche. ADO drives immunosuppression in solid tumors by binding to the A2BR on immune and tumor cells. Once activated, the A2BR increases the secretion of VEGF and IL-8 into the TME^[140,141]^. This secretion from immune cells comes mainly from monocyte-derived immune cells. When activated by ADO and A2BR interactions, these cells contribute major driving factors in tumor immunosuppression. Addi *et al.* discovered that the A2BR, not the A2AR, on bone marrow-derived DCs decreased the production of IL-12p70 in mice^[84]^. A2BR knock-out lung carcinoma cells produced lower VEGF levels in this model when stimulated with adenosine than wildtype controls^[140]^.

#### Hypoxia increases A2BR expression

As described earlier, hypoxia is a hallmark of solid tumors. Recent studies have expanded our understanding of the role of hypoxia in solid tumors, emphasizing its impact on angiogenesis and tumor growth and its influence on other aspects of tumor biology, such as immune evasion and therapy resistance^[18,142]^. Hypoxia-driven upregulation of A2BRs and ADO signaling contributes to tumor progression, angiogenesis, and immune escape^[44,113]^.

The hypoxic response also modulates the immune system, affecting innate and adaptive immunity. Hypoxia can alter the functions of immune cells such as TAMs, neutrophils, DCs, T cells, and NK cells^[18,143]^. Hypoxia-driven changes in the TME, for example, can create an immunosuppressive milieu, impairing the ability of immune cells to target and eliminate cancer cells^[144]^. Novel therapeutic strategies targeting hypoxia and adenosine signaling pathways, including A2BRs, are currently being investigated to improve the efficacy of existing cancer treatments and overcome treatment resistance^[14]^.

### The role of ADO and its receptors blockade to overcome resistance

ADO and its receptors are critical in maintaining immunosuppression in the TME, contributing significantly to immunotherapy resistance. Several clinical trials are exploring the potential of ADO receptor blockade as a novel strategy to counteract this resistance.

Several A2AR antagonists are currently being explored in clinical trials [Table 1], and two of the more advanced therapies are discussed further as part of this review: Corvus Pharmaceutical’s ciforadenant and AstraZeneca’s AZD4635^[145]^.

**Table 1:** List of current clinical trials evaluating A2AR and A2BR antagonists alone or in combination with cancer immunotherapies

**Drugs**	**Combinations**	**Clinical trial information**				
		**Phase**	**Indications**	**Enrollment**	**NCT number**	**Completion** **date**
EOS448 (A2AR antagonist)	· EOS-448, a small molecule, combined with pembrolizumab, an anti-PD-1 antibody · EOS-448 combined with inupadenant, an investigational adenosine A2AR antagonist · EOS-448 combined with dostarlimab, an anti-PD-1 antibody · Inupadenant combined with dostarlimab · EOS-448 combined with inupadenant and dostarlimab · EOS-448 combined with dostarlimab and standard-of-care chemotherapies in participants with NSCLC	1/2	Lung/H&N cancers/Melanoma	376	NCT05060432	2024-09
NIR178 (Taminadenant)	· DFF332, a small molecule targeted to HIF-2α · DFF332 in combination with everolimus, an mTOR inhibitor · DFF332 in combination with spartalizumab (an anti-PD-1 antibody) plus taminadenant (A2AR antagonist)	1	RCC	180	NCT04895748	2025-04
TT-10 (A2AR antagonist)	· TT-10, a small molecule, as a single agent	1/2	Prostate/NSCLC/RCC	90	NCT04969315	2025-08
ILB2109 (A2AR antagonist)	· ILB2109, a small molecule, as a single agent	1	Advanced solid tumor	48	NCT05278546	2024-01
AZD4635 (A2AR antagonist)	· AZD4635 as monotherapy · Combination with durvalumab · Combination with durvalumab plus oleclumab · Combination with docetaxel · Combination with either abiraterone acetate or enzalutamide	1	Solid tumor	313	NCT02740985	2021-04
AZD4635 (A2AR antagonist)	· AZD4635 with durvalumab, an anti-PDL-1 antibody · AZD4635 with oleclumab, an anti-CD73 antibody	2	Prostate tumor	59	NCT04089553	2023-04
CPI-444 (A2AR antagonist)	· CPI-444, a small molecule, in combination with ipilimumab, an anti-CTLA4 antibody · CPI-444 in combination with nivolumab, an anti-PD-1 antibody	1/2	RCC	15	NCT05501054	2026-11
CPI-444 (A2AR antagonist)	· CPI-444 (ciforadenant) as a single · Combination with atezolizumab, a PD-L1 inhibitor	1	RCC	502	NCT02655822	2021-07
PBF-1129 (A2BR antagonist)	· Combination of adenosine A2BR antagonist PBF-1129 (mAb) and nivolumab, an anti-PD-1 antibody	1	Metastatic NSCLC	30	NCT05234307	2025-12
	· PBF-1129 as a single agent	1	Metastatic NSCLC	18	NCT03274479	2023-12
M1069 (Dual A2AR/A2BR antagonist)	· M1069, a small molecule, as a single agent	1	Unresectable solid tumors	30	NCT05198349	2023-12
AB928 (Dual A2AR/A2BR antagonist)	· Combination of SRF617, an anti-CD39 antibody, etrumadenant (AB928), and zimberelimab (AB122), an anti-PD-1 antibody	2	Prostate cancer	15	NCT05177770	2023-04
TT-4 (A2BR antagonist)	· TT-4, a small molecule, as a single agent	1/2	GI cancers	69	NCT04976660	2023-09

Data obtained from ClinicalTrials.gov. A2AR: A2A receptors; A2BR: A2B receptors; CTLA4: T lymphocyte antigen 4; GI: gastrointestinal; H&N: Head & Neck; NSCLC: non-small cell lung cancer; RCC: renal cell carcinoma.

In a first-in-human Phase 1 dose-escalation study in patients with advanced refractory cancers (NCT02655822), ciforadenant (either monotherapy or in combination with atezolizumab) was administered to 502 patients. Of those, a cohort of 68 renal cell carcinoma (RCC) patients yielded clinical responses, including partial responses (PR) in 11% of patients treated with a combination of A2AR antagonists and anti-PD-L1 antibodies, and in 3% of patients treated with A2AR antagonists alone^[146]^. Further, tumor regression was observed in an additional 24% of patients, although the regression was not significant enough to be classified as PR by RECIST criteria. These findings are noteworthy, especially considering that the patients involved in the study were not only resistant to PD-1 blockade but were also deemed untreatable prior to the trial.

According to Michail Sitkovsky^[147]^, the observed tumor regressions in patients with RCC, who were previously untreatable and refractory to PD-1 blockade, likely occurred in patients meeting specific criteria: their tumors were immunogenic, developed tumor-reactive effector T cells, retained a significant number of effector cells post-toxic cancer chemotherapies, and were protected by immunosuppressive extracellular ADO to A2AR signaling. Ciforadenant appears to have facilitated the invasion and tumor-rejecting functions of T and NK cells in these patients; however, the levels of antitumor immunity in responsive patients were not high enough to achieve a complete response. The major limitation appears to be the lack or low numbers of tumor-reactive T and NK cells in refractory patients, either due to the tumor’s poor immunogenicity or past toxic chemotherapies.

A2AR antagonists are anticipated to be most efficacious in patients with sufficient aggressive, multifunctional tumor-reactive T cells. Without these cells, it could be expected that A2AR antagonists would only have antitumor effects when combined with cancer vaccines or T-cell transfers that increase the number of tumor-reactive T cells. Future treatments combining A2AR antagonism with adoptive cell transfer (ACT) are promising, especially for refractory patients, as ACT ensures the presence of sufficient T-cells and NK-cells in patients, enhancing the potential for A2AR antagonism as an immunotherapy.

These antagonists have been able to show, both *in vitro* and *in vivo*, that blocking the adenosine pathway at the A2AR increases cytotoxic T cells within the TME, increases cytokine production, and reverses T cell inhibition.

Another A2AR antagonist in development is AstraZeneca’s AZD4635. In a monotherapy phase 1 trial (NCT02740985), observed adverse events included nausea, fatigue, and vomiting. In addition, one patient with colorectal cancer had sudden death 15 days after the last dose of AZD4635, which was considered treatment-related by the investigator. However, AZD4635 was well tolerated both as a monotherapy and in combination with durvalumab in all patients. There were patients with responses such as stable disease, partial response, and complete response and RNA analysis confirmed that in 5 of 7 patients, intertumoral adenosine signaling decreased. Four of these 7 patients also had increases in gene-expression signatures of cytolytic activity and IFN-γ signaling. These findings suggest that there were observable positive responses to the treatment in some patients.

A2BR antagonists are also under investigation in several clinical trials. Arcus’ etrumadenant, a dual A2AR and A2BR antagonist, is being evaluated in several cancers and was recently discontinued in mCRPC due to a lack of efficacy (NCT05177770). Palofarma’s PBF-1129, an A2BR antagonist, is being evaluated in metastatic NSCLC (NCT03274479) and EMD Serono’s M1069 (NCT05198349), another A2AR and A2BR antagonist, is currently undergoing a first-in-human trial in patients with advanced malignancies. In addition, Portage is evaluating TT-4, an A2BR antagonist, as a single agent in gastrointestinal cancer (NCT04976660). Further investigation will likely provide more insights into the clinical potential of these promising strategies. Overall, compounds exhibiting the highest water solubility tend to possess increased bioavailability, making them more effective^[148]^. With the advancement in the development of ADO receptor antagonists, enhancing the solubility of these promising compounds while preserving their selectivity emerges as an avenue for improvement.

## CONCLUSION

In the complex battleground of cancer, it is necessary to understand the adaptations tumors employ to resist therapies. This review has emphasized the role of ADO, a significant player in the TME, in driving immunosuppression and fostering cancer drug resistance. The importance of ADO and its receptors, particularly the A2AR and A2BR subtypes, in promoting an immune-escaping environment was thoroughly explored.

Current research endeavors focus on various approaches to counteract immunosuppression, including monoclonal antibodies against CD73 and the blockade of ADO receptors. Ongoing clinical trials investigate combinations of these approaches with existing therapies, aiming to stimulate immune responses and improve patient outcomes.

The results of ongoing clinical trials will inform new ways of overcoming cancer drug resistance. However, further research is required to understand and fully exploit ADO’s pathway. Targeting ADO could improve cancer treatments, providing hope for patients who previously had limited treatment options. This underscores the importance of ongoing research in this area, aiming to improve the prognosis for all cancer patients.

## DECLARATIONS

### Copyright

© The Author(s) 2023.
